# Video-Laryngoscopy-Assisted Fishbone Removal from the Upper Digestive Tract; a Letter to the Editor

**DOI:** 10.22037/aaem.v9i1.1068

**Published:** 2021-01-05

**Authors:** Petros V. Vlastarakos, Konstantinos Chondrogiannis

**Affiliations:** 1 ENT Department, MITERA Infirmary, Athens, Greece.; 2 Anaesthesiology Department, MITERA Infirmary, Athens, Greece.

**Keywords:** Laryngoscopy, video-assisted surgery, hypopharynx, fishes, foreign bodies

**Dear Editor**

Fish bones are frequently lodged in the upper digestive tract, usually at the palatine tonsils, tongue base, valleculae, and pyriform sinuses. The otorhinolaryngologist represents the first point of contact in such cases, which may in fact account for a sizeable percentage of ENT emergencies (1). Persistent sharp pain in the throat, experienced by the patient following eating fish, indicates that a fishbone has stuck. If the bone is not removed in a timely manner, it may result in serious septic complications (2).

Fishbone removal requires dexterity on the part of the ENT Surgeon and co-operation on the part of the patient. Removal of fishbones in the oro-pharynx or base of tongue is usually easy; bones lodged further down may require a three-hand technique, i.e. the patient or an assistant holding the tongue, and not infrequently, turn out to be an intolerable task in the outpatient setting. We Have succeeded in managing such cases under general anaesthesia without intubation, with the use of a rigid anaesthetic video-laryngoscope and a pair of Magill forceps.

Placed in supine position, the patient is pre-oxygenated. Induction to anesthesia with propofol and remifentanil is followed by ventilation. The anaesthetist elevates the vallecular aspect of the epiglottis, using a blade with a steep curved bend for alignment of the oral, pharyngeal, and laryngeal axes, to fully expose and visualize the glottis and adjacent areas of the hypopharynx. With the video image projected from the distal end of the laryngoscope blade, the ENT Surgeon advances the Magill forceps (or any rigid instrument felt appropriate) until the fishbone is grasped. The bone is subsequently removed with a slight rotating movement. The two-dimensional visualization of a three-dimensional area and the need to interrupt for ventilation, require communication and co-ordination between the Anaesthetist and the ENT Surgeon, but the respective learning curve is fairly short.

The advantages include good illumination, clear visualization, and precise extraction (3). The technique itself is efficient, safe, well tolerated, and with low morbidity, being analogous with the concept of the “four hands technique” employed in various endoscopic surgeries (4). Despite the limited indication, this method may reduce limitations associated with non-invasive fishbone removal, the ingestion of which would most likely continue to accompany the eating habits of human societies.

**Figure 1:**
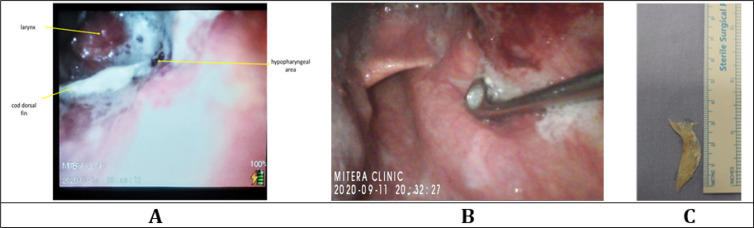
**A:** The dorsal fin of a cod horizontally lodged in the area of the pyriform sinuses. **B:** Fishbone grasped with a pair of Magill forceps under video-laryngoscopic guidance (different case than figure 1). **C:** 3cm fishbone removed en-bloc from the hypopharynx via the video-laryngoscopy-assisted technique

## Conflicts of interest

None declared. 

## Funding

The authors have no financial interest and have not received any financial support for this article.

## Ethical approval

All procedures performed were in accordance with the ethical standards of the institutional and national research committee and with the 1964 Helsinki declaration and its later amendments. The patients signed a consent form regarding the publication of intra-operative photographs for educational or other purposes.
